# TFAP2A drives non-small cell lung cancer (NSCLC) progression and resistance to targeted therapy by facilitating the ESR2-mediated MAPK pathway

**DOI:** 10.1038/s41420-024-02251-5

**Published:** 2024-12-18

**Authors:** Ding-Guo Wang, Jian Gao, Jing Wang, Kun-Chao Li, Zhi-Bo Wu, Zhong-Min Liao, Yong-Bing Wu

**Affiliations:** 1 Department of Cardiothoracic Surgery, The Second Affiliated Hospital, Jiangxi Medical College, Nanchang University, Nanchang, China; 2 Department of Thoracic Surgery, Zhongshan Hospital, Fudan University, Shanghai, China

**Keywords:** Non-small-cell lung cancer, Cancer therapy

## Abstract

Cancer is among the leading causes of death related diseases worldwide, and lung cancer has the highest mortality rate in the world. Transcription factors (TFs) constitute a class of structurally and functionally intricate proteins. Aberrant expression or functional deficiencies of transcription factors may give rise to abnormal gene expression, contributing to various diseases, including tumours. In this study, we propose to elucidate the potential role and mechanism of TFAP2A in NSCLC. We found that TFAP2A levels were significantly greater in tumour tissues than para-tumour tissues, and high expression of TFAP2A was associated with poor prognosis in NSCLC patients. Additionally, TFAP2A overexpression promoted NSCLC progression both in vivo and in vitro. Mechanistically, ESR2 is a potential target regulated by TFAP2A and that TFAP2A can bind to the promoter region of ESR2. Furthermore, the overexpression of both TFAP2A and ESR2 in NSCLC cells was associated with the overactivation of MAPK signalling, and the combination of PHTPP and osimertinib had a synergistic effect on suppressing tumour growth.

## Introduction

Lung cancer is one of the most prevalent malignancies, and lung cancer can be categorised into two primary histological types according to pathology, namely, small cell lung cancer (SCLC, accounting for approximately 15%) and non-small cell lung cancer (NSCLC, accounting for approximately 85%). NSCLC can be further divided into lung adenocarcinoma (LUAD), lung squamous cell carcinoma (LUSC), large cell carcinoma, adenosquamous carcinoma, and other less common histotypes [1]. The prognosis of NSCLC patients diagnosed at stage I has remained similar to that in the past, reaching approximately 80%. Notably, the 5-year survival rate of some advanced NSCLC patients and tumour metastasis patients has significantly increased with advancements in tumour diagnosis and therapeutic strategies. However, NSCLC poses a substantial challenge due to the absence of prominent symptoms during its early stages, resulting in delayed diagnosis for the majority of patients. Consequently, the overall prognosis for NSCLC patients remains poor, with a mere 26% 5-year survival rate [2–4].

Transcription factors (TFs) usually contain a DNA-binding domain (DBD) that can bind to a specific sequence on DNA, thereby affecting gene transcription. Abnormal expression or functional defects of transcription factors may lead to abnormal gene expression, thereby causing various diseases, such as tumours, cardiovascular disease and neurological disease [5–7]. Members of the transcription factor activator protein-2 (TFAP-2) family (TFAP2A, TFAP2B, TFAP2C, TFAP2D and TFAP2E) are associated with various pathophysiological processes, including tumours. For example, TFAP2A has been reported to reduce the expression of CRTAC1 by promoting TPRG1-AS1 transcription, thereby accelerating glycolysis and angiogenesis in bladder urothelial carcinoma (BLCA) and promoting the progression of BLCA [8]. In lung cancer, TFAP2A drives cancer progression through multiple pathways. For example, TFAP2A enhances the metastatic ability of lung adenocarcinoma through the miR-16/TFAP2A/PSG9/TGF-β axis [9] and promotes the progression of lung adenocarcinoma through epithelial–mesenchymal transition (EMT) by inducing the expression of KRT16 [10]. USP22 promotes the invasion of NSCLC in a TFAP2A-dependent manner [11]. Thus, TFAP2A plays an important role in the development of tumours [12, 13].

Oestrogen plays a significant role in the initiation and progression of diverse malignancies, including breast cancer, ovarian cancer, prostate cancer and colon cancer. Oestrogen was primarily discovered to exert its effects on cellular activity through the activation of two distinct subtypes of oestrogen receptors, namely, oestrogen receptor (ER) alpha (ESR1) and ER beta (ESR2) [14]. The oestrogen receptor, a member of the nuclear membrane receptor family, mostly localises to the nuclear membrane, with little expression in the cytoplasm and mitochondria. ESR2 has a diverse range of behaviours and can modulate the expression of genes associated with signal transduction, cell cycle progression and apoptosis. Previous studies have shown that ESR2 is involved in the progression of NSCLC and is the predominant form in lung cancer [15, 16]. For example, oestrogen receptors upregulate OPN expression and promote lung cancer cell migration via the MAPK pathway [17]. In addition, combined targeting of the ER and EGFR in NSCLC has demonstrated enhanced antiproliferative effects [18].

Osimertinib is a mutant-selective EGFR tyrosine kinase inhibitor, and NSCLC patients with a positive EGFR T790M mutation who have locally progressed or metastatic disease respond well to therapy [19]. The use of osimertinib in treating EGFR-mutated NSCLC inevitably results in acquired resistance, thus diminishing its therapeutic effectiveness. The mechanisms driving this resistance are complex and include both EGFR-dependent and EGFR-independent pathways. To overcome this obstacle, several therapeutic approaches have been developed for NSCLC patients who develop resistance to osimertinib [20, 21]. In the present study, TFAP2A was discovered to be highly expressed in NSCLC, and the oncogenic role of TFAP2A in NSCLC was verified in vivo and in vitro. Furthermore, ESR2 was identified as the transcriptional target of TFAP2A by bioinformatics analysis and ChIP, and the combination of the ESR2 inhibitor PHTPP and osimertinib had a synergistic effect on NSCLC with a high level of TFAP2A. Thus, these findings identified the fundamental molecular pathways and highlighted the prospective therapeutic applications of TFAP2A in forthcoming therapies for NSCLC.

## Results

### TFAP2A is highly expressed in NSCLC

**Fig. 1:**
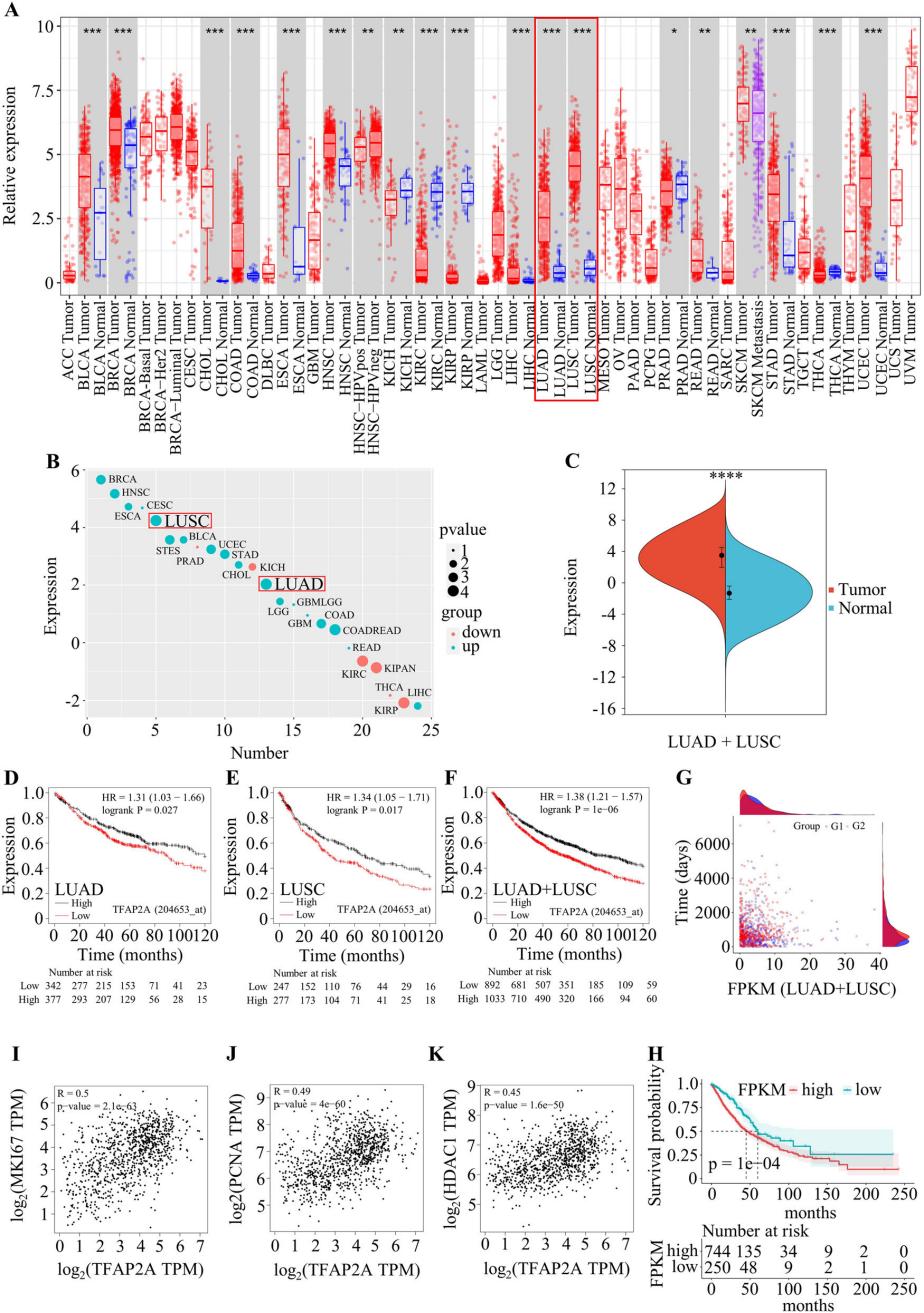
Expression level and prognosis of TFAP2A. **A** TFAP2A levels in different tumours. **B** Bubble plot of expression scores for various malignant tumours. **C** TFAP2A levels in NSCLC samples according to the Sangerbox 3.0 database. **D**–**H** OS of patients with NSCLC from different databases. **I–K** Relationships between TFAP2A and proliferation markers according to the GEPIA 2 database.

Previous studies based on public databases have shown that TFAP2A is highly expressed in tumours [12, 13, 22]. However, the role of TFAP2A in NSCLC has not been further investigated. The present study demonstrated that TFAP2A was highly expressed in different tumours, including LUAD and LUSC, according to the TIMER2.0 database (Fig. 1A). A bubble plot showed that TFAP2A was a potential factor for the occurrence of LUSC and LUAD (Fig. 1B), and the level of TFAP2A was significantly greater in LUAD and LUSC tissues than in normal tissues (Fig. 1C). Analysis of the overall survival of LUAD and LUSC patients based on the Kaplan‒Meier plotter database indicated that elevated levels of TFAP2A were associated with poor prognosis in LUAD and LUSC patients (Fig. 1D–F). Similar results were obtained in scatterplots generated from these available data and the Sangerbox 3.0 database (Fig. 1G, H). To explore the relationship between TFAP2A and tumour cell viability, the coexpression of TFAP2A with Ki67, PCNA and HDAC1 was analysed. The levels of these proliferative markers were positively related to the level of TFAP2A (Fig. 1I–K). Thus, these findings indicated that TFAP2A is a potential risk factor for NSCLC and that TFAP2A may promote tumour growth.

### Increased level of TFAP2A is correlated with an unfavourable prognosis in NSCLC patients

**Fig. 2:**
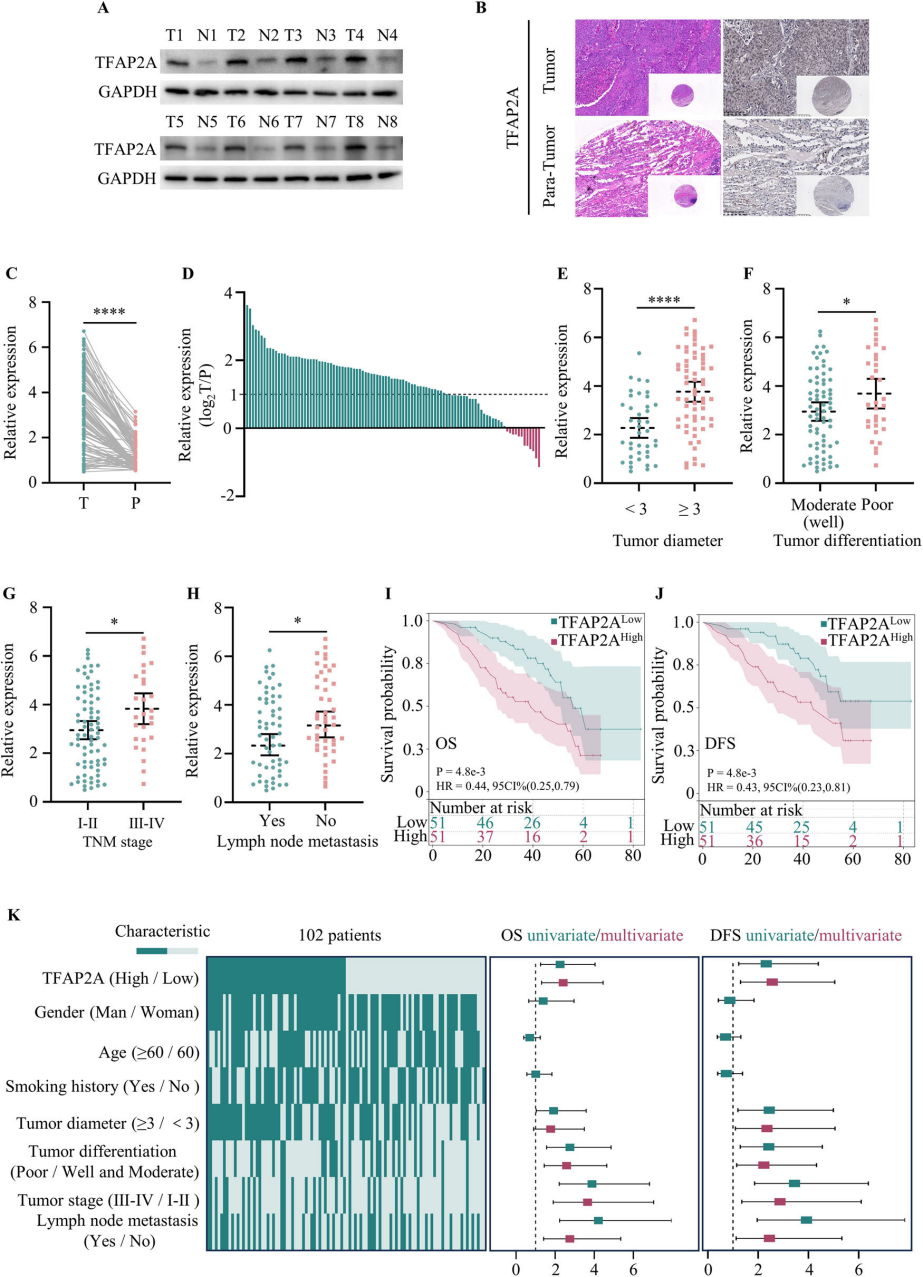
TFAP2A upregulation in NSCLC patients predicts poor prognosis. **A**, **B** Western blot analyses and IHC images of NSCLC tissues. **C**, **D** The level of TFAP2A in tumours and para-tumours was measured by qRT‒PCR. **E**–**H** Statistical analysis of TFAP2A expression in relation to tumour size, tumour differentiation, TNM stage and lymph node metastasis. **I**, **J** OS and DFS of patients in the TFAP2A^High^ and TFAP2A^Low^ groups with NSCLC. **K** Univariate and multivariate analyses of factors associated with OS and DFS.

To validate the level of TFAP2A in NSCLC, IHC and Western blot analyses was conducted to measure TFAP2A expression in tumour tissues, which demonstrated that the level of TFAP2A was higher in tumour tissues than in para-tumour tissues (Fig. 2A, B). qRT‒PCR analysis of 102 paired tumour and para-tumour tissues indicated that the mRNA level of TFAP2A was significantly elevated in tumour tissues, and the TFAP2A level was twofold greater in 80% of paired tissues (Fig. 2C, D). In addition, the associations between TFAP2A expression and clinical characteristics were evaluated in 102 patients. The protein level of TFAP2A was positively related to tumour diameter, tumour differentiation, TNM stage, and lymph node metastasis (Fig. 2E–H). Furthermore, the overall survival (OS) and disease-free survival (DFS) results showed that patients with high levels of TFAP2A had a poor prognosis (Fig. 2I, J). In addition, univariate and multivariate analyses of OS revealed that TFAP2A level, tumour differentiation, tumour stage and lymph node metastasis were independent risk factors. Moreover, TFAP2A level, tumour diameter, tumour differentiation, tumour stage and lymph node metastasis were independent risk factors related to DFS (Fig. 2K). In summary, these findings demonstrated that TFAP2A levels are elevated in tumour tissues compared to para-tumour tissues and are associated with poor prognosis of NSCLC patients.

### Elevated TFAP2A enhances the proliferation, migration, and invasion of NSCLC cells

**Fig. 3:**
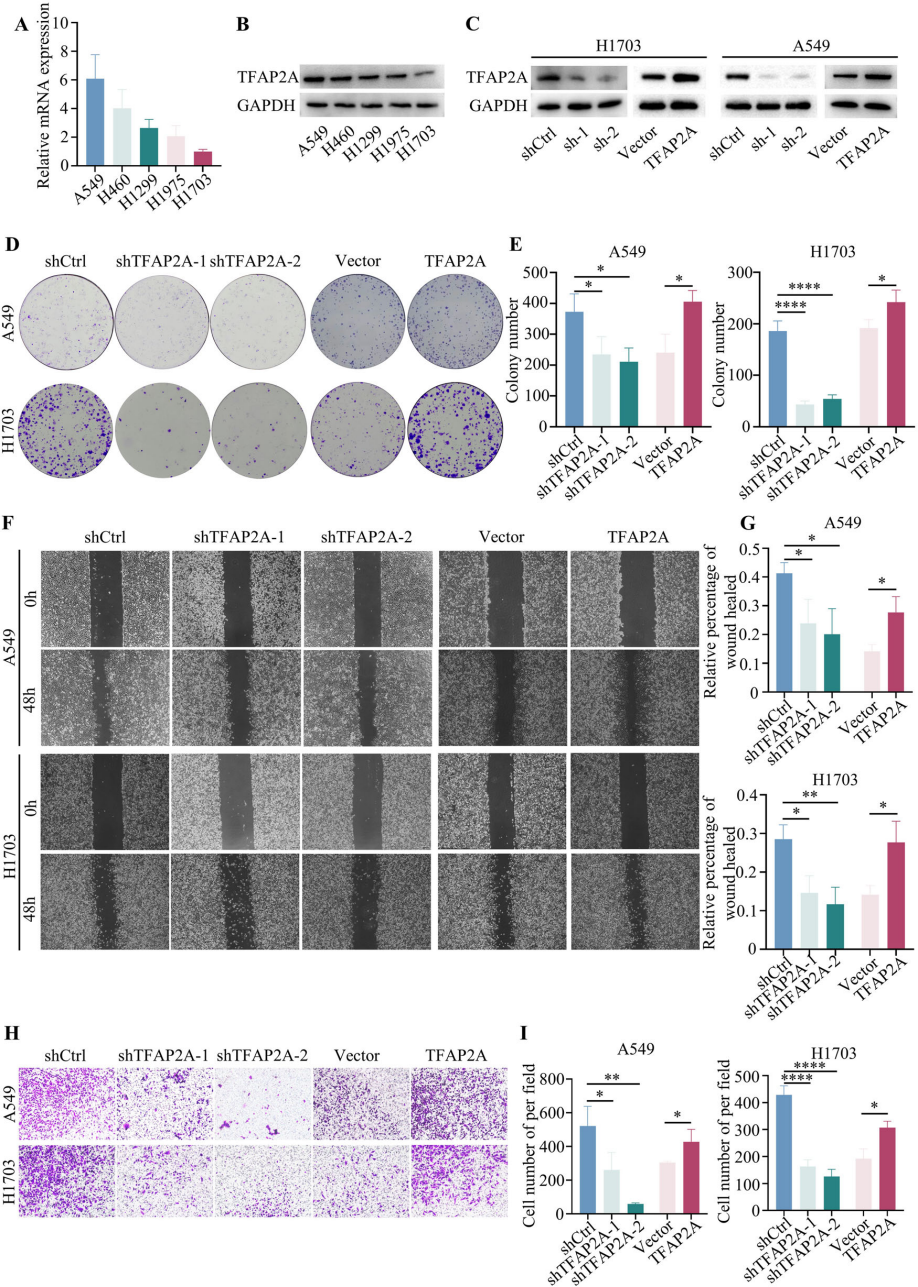
TFAP2A promotes NSCLC proliferation, invasion, and migration. **A**, **B** The mRNA and protein levels of TFAP2A were measured in five NSCLC cell lines. **C** The overexpression and knockdown efficiencies were assessed by Western blot analysis. **D**, **E** The proliferation of TFAP2A-overexpressing and TFAP2A-knockdown tumour cells was assessed by colony formation assays. **F**–**I** The invasion and migration abilities of TFAP2A-overexpressing and TFAP2A-knockdown tumour cells were evaluated by wound-healing and Transwell assays.

qRT‒PCR and Western blot analyses were conducted to assess the level of TFAP2A in five NSCLC cell lines, which demonstrated that the relative level of TFAP2A gradually increased among the A549, H1299, H460, H1975 and H1703 cells (Fig. 3A–B). Therefore, A549 and H1703 cells were selected for TFAP2A overexpression and knockdown experiments, and shRNA-1/shRNA-3 had a significant effect on the three tested shRNAs. The overexpression and knockdown efficiencies were confirmed by Western blotting (Fig. 3C). Colony formation assays of A549 and H1703 cells showed that TFAP2A overexpression promoted the growth of tumour cells and that TFAP2A knockdown inhibited tumour cell growth (Fig. 3D, E). The invasion and migration abilities were enhanced by TFAP2A overexpression, but TFAP2A knockdown inhibited these abilities (Fig. 3F–I). Thus, these findings indicated that high levels of TFAP2A promote the progression of NSCLC cells.

### TFAP2A promotes NSCLC growth and is associated with poor prognosis in vivo

**Fig. 4:**
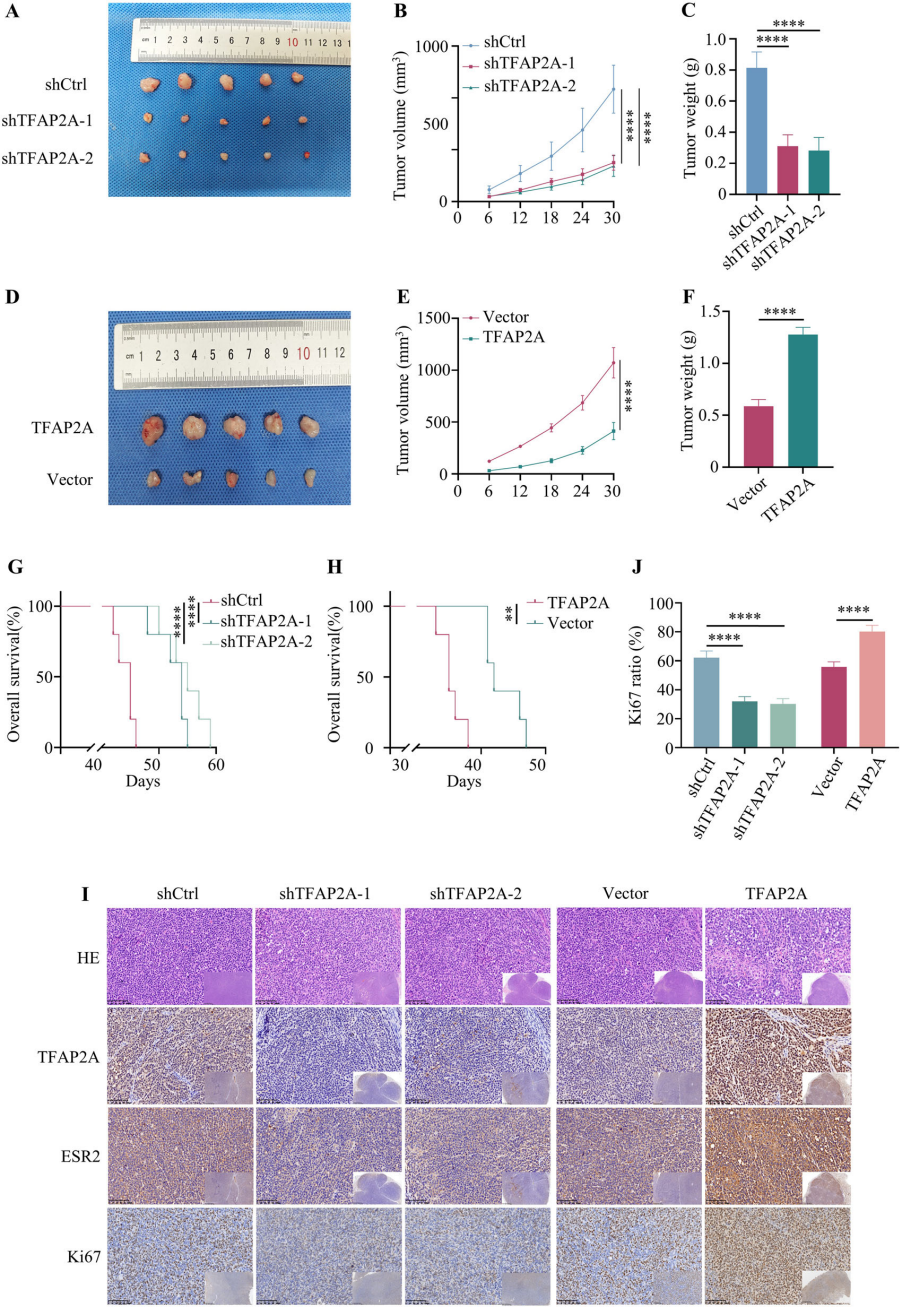
Oncogenic role of TFAP2A in vivo. **A**–**C** TFAP2A knockdown inhibited tumour growth. **D**–**F** The tumour size and weight were changed after TFAP2A overexpression. **G**, **H** Survival of mice with TFAP2A overexpression or knockdown. **I**, **J** IHC results of subcutaneous tumours, and the Ki67 ratio of each group was analysed by ImageJ.

A subcutaneous tumour model in nude mice was constructed to further verify the oncogenic role of TFAP2A. TFAP2A knockdown decreased the tumour size and inhibited tumour growth (Fig. 4A–C). However, the tumours in nude mice injected with A549-TFAP2A cells were larger and grew faster than those in mice injected with A549-vector cells (Fig. 4D–F). Moreover, TFAP2A knockdown decreased survival, whereas TFAP2A overexpression increased survival (Fig. 4G, H). The subcutaneous tumours were analysed by IHC, which demonstrated that the expression levels of TFAP2A, ESR2 and Ki67 were decreased in tumours derived from TFAP2A-knockdown cells but increased in tumours derived from TFAP2A-overexpressing cells (Fig. 4I, J). These results revealed the carcinogenic role of TFAP2A in NSCLC.

### TFAP2A-mediated transcriptional activation of ESR2

**Fig. 5:**
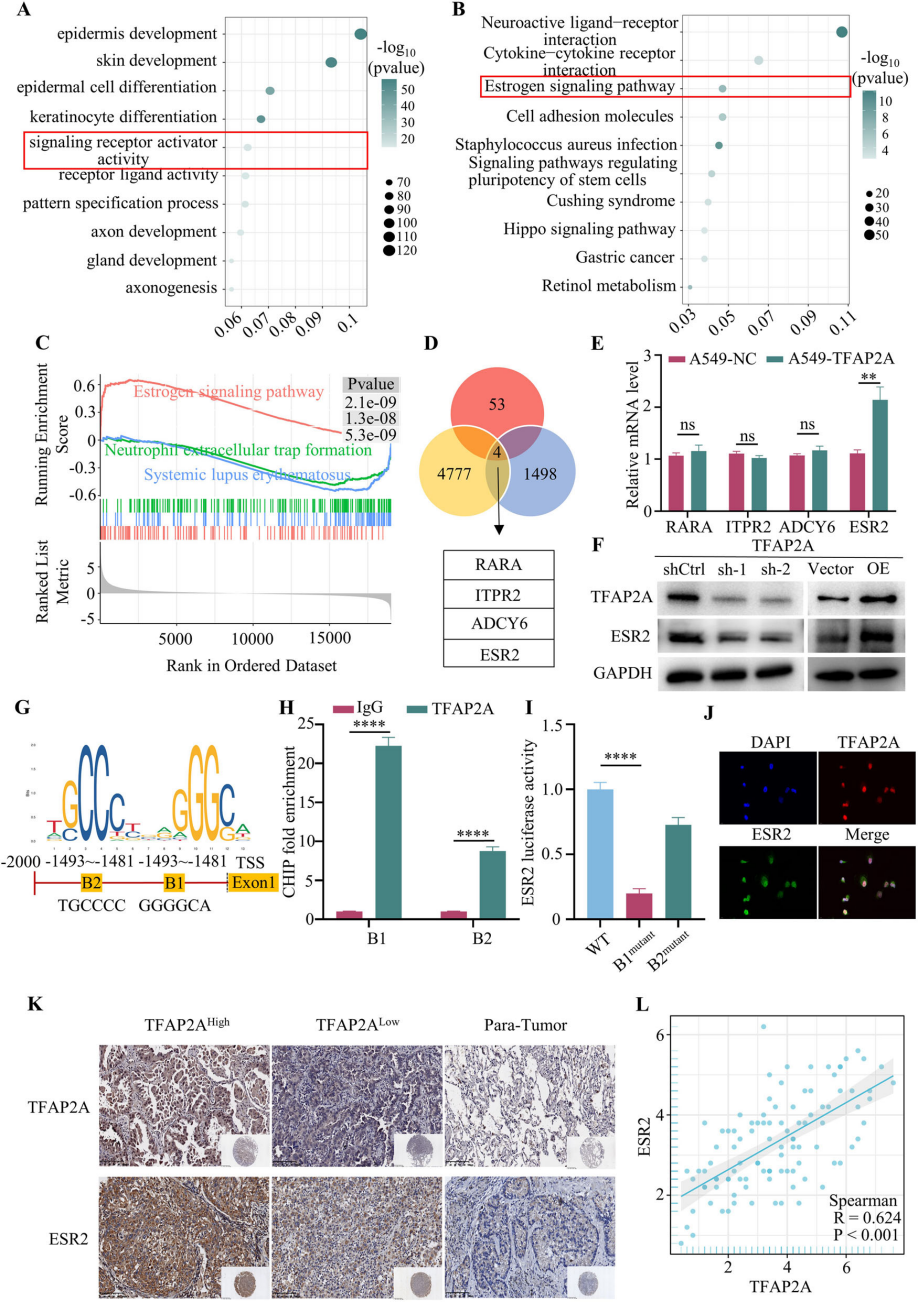
TFAP2A-mediated transcriptional activation of ESR2. **A**–**C** GO, KEGG and GSEA results based on TCGA data. **D** The genes involved in the oestrogen signalling pathway were downloaded from KEGG, and the targets of TFAP2A were obtained from the JASPAR and hTFtarget databases. Venn diagram showing the potential targets of TFAP2A in the oestrogen signalling pathway. **E** qRT‒PCR analysis of the four anticipated targets in A549-vector and A549-TFAP2A cells revealed a significant alteration ESR2 expression. **F** The relative levels of TFAP2A and ESR2 after TFAP2A overexpression or knockdown. **G** The binding motif of TFAP2A was identified, and two potential binding sites within the promoter region of ESR2 were identified. **H** A ChIP assay was conducted to confirm the binding site of ESR2 within the TFAP2A promoter region. **I** A luciferase assay was performed to confirm the binding of ESR2 to the three putative regions of TFAP2A. **J** The localisation of TFAP2A and ESR2 was shown by IF. **K**, **L** IHC images of TFAP2A and ESR2 in tumour and para-tumour tissues, and the level of TFAP2A was positively related to the ESR2 level.

To conduct a more comprehensive investigation into the mechanisms by which TFAP2A facilitates NSCLC progression, potential transcriptional targets of TFAP2A were predicted using bioinformatics analysis. LUAD and LUSC data were downloaded from TCGA database, and all patients were divided into two groups (TFAP2A^high^ vs. TFAP2A^Low^) based on the median level of TFAP2A. The DEGs between the TFAP2A^high^ and TFAP2A^Low^ groups were analysed. All the upregulated genes (*P* < 0.05) were subjected to GO and KEGG analyses, and the top ten terms are presented in Fig. 5A, B. Furthermore, all differentially expressed genes were subjected to GSEA, which indicated that the oestrogen signalling pathway had the highest score among all pathways (Fig. 5C). Based on these results, the oestrogen signalling pathway may play an important role in the promotion of tumour progression by TFAP2A. To further explore the potential targets of TFAP2A, the transcriptional genes were predicted utilising the JASPAR and hTF target databases, and the oestrogen signalling pathway genes identified by KEGG analysis were downloaded. The Venn diagram shows the four potential targets of TFAP2A in the oestrogen signalling pathway (Fig. 5D). qRT‒PCR was performed to evaluate the relative mRNA levels of RARA, ITPR2, ADCY6 and ESR2 in the A549-vector and A549-TFAP2A cells, which identified ESR2 as the only target (Fig. 5E). In addition, the protein level of ESR2 decreased with TFAP2A knockdown but increased with TFAP2A overexpression (Fig. 5F).

The TFAP2A binding motif was identified using JASPAR, and the top two potential binding sites (B1 and B2) are shown in Fig. 5G. A ChIP assay showed that B1 had a stronger binding affinity for TFAP2A (Fig. 5H). Vectors containing mutations in the two binding sites (GGGC to AAGC and GCCC to AACC) were designed, generated, and transfected into HEK293T cells. There was a substantial decrease in luciferase activity after transfection of the B1 mutant vectors compared to WT vectors (Fig. 5I). Consistently, the interaction between the two proteins in A549 cells was verified by IF (Fig. 5J). In addition, the TMA IHC scores revealed that TFAP2A was positively correlated with ESR2 (Fig. 5K, L). Taken together, these results showed that TFAP2A targets ESR2 and activates its transcription.

### Targeting TFAP2A or ESR2 sensitises NSCLC cells with high TFAP2A expression to osimertinib-targeted therapy

**Fig. 6:**
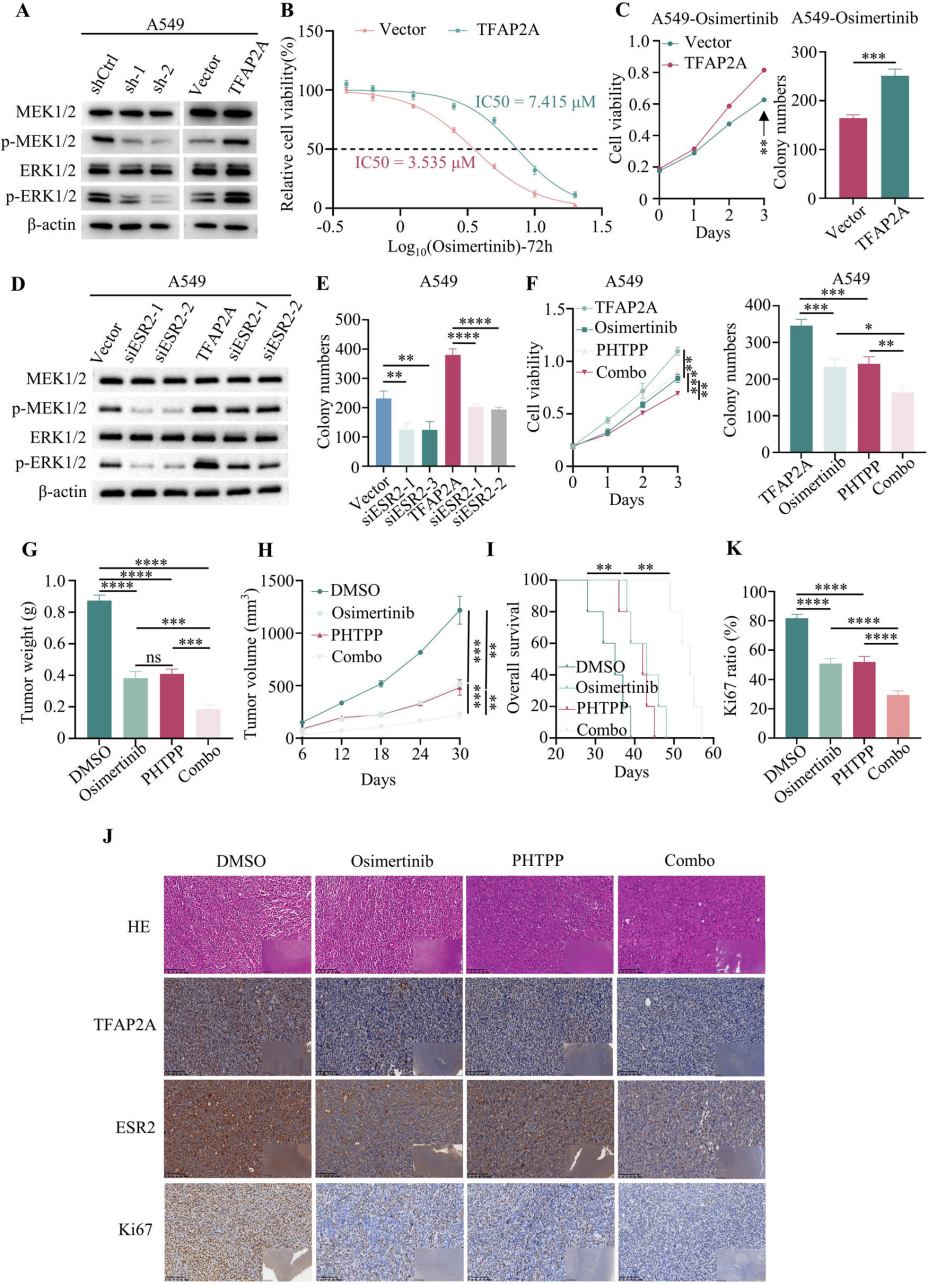
Targeting TFAP2A/ESR2 signals sensitises NSCLC cells with high TFAP2A expression to osimertinib-targeted therapy. **A** The levels of p-MEK1/2 and p-ERK1/2 were decreased after TFAP2A knockdown but increased after TFAP2A overexpression. **B** IC50 of osimertinib in A549 cells after 48 h of treatment. **C** CCK-8 and colony formation assays showed that TFAP2A knockdown sensitised cells to osimertinib, while overexpression of TFAP2A conferred resistance to osimertinib. **D**, **E** The p-MEK1/2 and p-ERK1/2 levels were decreased after ESR2 knockdown, and ESR2 knockdown inhibited the viability of NSCLC cells. **F** Effect of osimertinib, PHTPP, and osimertinib + PHTPP on tumour cell proliferation. **G**, **H** Dual blockade of TPHPP and targeted therapy markedly decreased tumour size and decreased tumour growth. **I** Combining TPHPP with osimertinib significantly prolonged the survival of mice. **J** IHC staining of TFAP2A, ESR2 and Ki67 in subcutaneous tumours after drug administration. **K** Ki67 ratio in the DMSO, Osimertinib, PHTTP and Combo group.

Previous studies have reported that ESR2 promotes tumour progression by activating MAPK signalling [23, 24]. Therefore, the present study assessed whether high levels of TFAP2A and/or ESR2 promote NSCLC progression via the MAPK signalling pathway. The levels of p-MEK1/2 and p-ERK1/2 decreased after TFAP2A knockdown but increased with TFAP2A overexpression (Fig. 6A). A previous study has revealed that the MAPK pathway may play a critical role in NSCLC associated with acquired resistance to osimertinib therapy [25]. Given the apparent involvement of TFAP2A in the MAPK pathway, the impact of TFAP2A overexpression on the responsiveness of tumour cells to osimertinib, a first-line targeted drug for NSCLC patients, was investigated. The IC50 values of A549-vector and A549-TFAP2A cells were 3.535 and 7.415 μM, respectively (Fig. 6B), and osimertinib reduce MEK and ERK phosphorylation with the dosage adopted (Supplementary Fig. 1A). The result shows that TFAP2A overexpression promoted the resistance of tumour cells to osimertinib (Fig. 6C). Moreover, ESR2 knockdown significantly impacted the levels of p-MEK1/2 and p-ERK1/2 in cells without TFAP2A overexpression, suggesting that the overexpression of TFAP2A is sufficient to interfere with the regulatory influence of ESR2 (Fig. 6D). The colony formation assay showed that the proliferation of TFAP2A-overexpressing and control tumour cells decreased when ESR2 was knocked down (Fig. 6E). PHTPP is a selective oestrogen receptor β antagonist, and an in vitro assay revealed that the combination of osimertinib and PHTPP had a greater effect than that of PHTPP or osimertinib alone in TFAP2A-overexpressing cells (Fig. 6F).

**Fig. 7:**
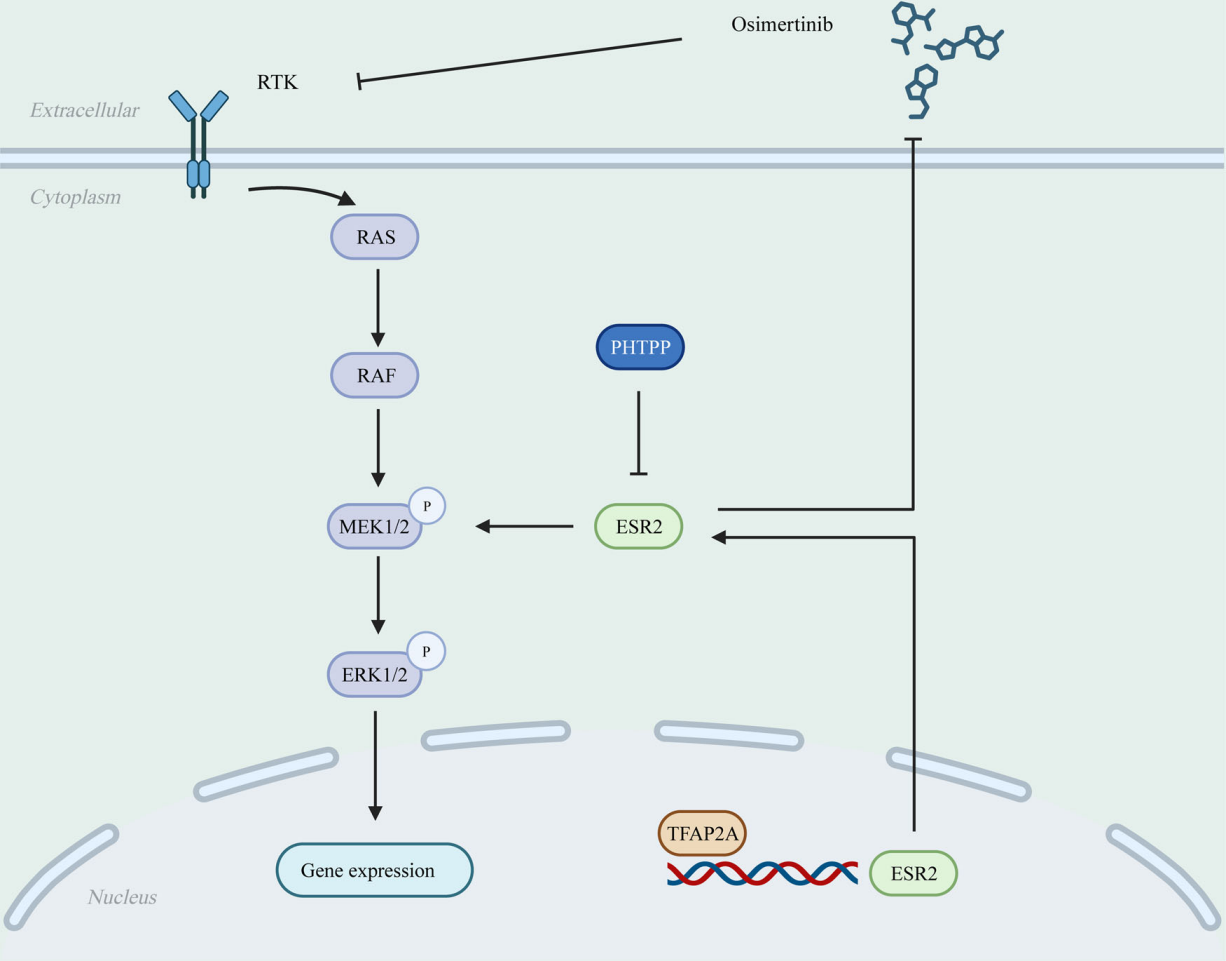
Diagram of the mechanism by which TFAP2A regulates NSCLC growth and progression. In NSCLC, TFAP2A targets ESR2 and activates the transcription of ESR2, and the ESR2-encoded protein, ESR2, activates MAPK signalling thereby promoting NSCLC growth and progression.

To further verify the synergistic effect of osimertinib and PHTPP in NSCLC, in vivo experiments were conducted. The combination of osimertinib and PHTPP significantly decreased the tumour size and inhibited tumour growth (Fig. 6G, H). Moreover, combining osimertinib with PHTPP significantly increased the survival of mice (Fig. 6I). In addition, the mice experience no toxicity with these dosages, and the weight of different groups mice showed no significance (Supplementary Fig. 1B). The IHC results (Fig. 6J) showed that the Ki67 ratio was decreased in the combination group compared to the other groups (Fig. 6K). Thus, these findings suggested that high expression of TFAP2A and ESR2 may influence targeted therapy and that PHTPP may sensitise NSCLC to high TFAP2A expression (Fig. 7).

## Discussion

In the present study, bioinformatics analysis indicated that the level of TFAP2A in NSCLC patients was significantly elevated, and a high level of TFAP2A was associated with poor prognosis. In addition, the TFAP2A level was positively related to important proliferative markers (Ki67, PCNA and HDNC1), which suggested that TFAP2A may promote the proliferation of tumours. The clinical data revealed that a high level of TFAP2A was associated with increased tumour size, poor tumour differentiation, advanced TNM stage, and lymph node metastasis. Patients with high TFAP2A expression had shorter survival times, and TFAP2A was a risk factor for OS and DFS. The potential targets (ESR2) of TFAP2A were predicted through GSEA and analysis of a public database. Given the synergistic effect of ESR2 in targeted therapy of NSCLC, the present study demonstrated that the combination of the ESR2 inhibitor TPHPP and osimertinib had a greater influence on tumour progression via in vivo and in vitro assays. The above results revealed that TFAP2A targets ESR2 and activates the MAPK signalling pathway, suggesting that inhibiting ESR2 may sensitise NSCLC cells to targeted therapy.

The incidence of lung cancer is second in the world, while the fatality rate of lung cancer is the highest, with approximately 2.2 million new cases and 1.79 million deaths every year. China has the highest lung cancer incidence and mortality rate (37.0% and 39.8%, respectively) in the world. According to the latest cancer statistics released by the National Cancer Centre, there were 4.064 million new cases in China in 2016, with an incidence rate of 186.46/100,000, 2.414 million new deaths, and a mortality rate of 1.0519/100,000. Among them, there were 828,000 new cases of lung cancer, with an incidence rate of 36.46/100,000, 657,000 new deaths, and a death rate of 28.09/100,000, ranking first among other tumours [26]. Targeted therapies have been rapidly developed over the past two decades. Osimertinib is a third-generation tyrosine kinase inhibitor (TKI) that targets the EGFR receptor. Although TKI drugs have been developed [19], resistance to targeted medications remains unavoidable. Therefore, the investigation of strategies to enhance the efficacy of targeted medications by improving their sensitivity to tumours is important [27].

TFs bind to a specific DNA sequence and form a complex with other proteins to increase or block the recruitment of specific genes to RNA polymerase, thereby regulating gene expression. Hence, the regulation of target genes by transcription factors can impact tumour growth. However, the precise involvement of the TFAP-2 gene family in the process of carcinogenesis remains uncertain. Several articles on the role of TFAP2A/B/C in lung cancer progression have been published, but the role of TFAP2D/E in this process is still unknown. Jeffrey R et al. reported that TFAP2α promotes gene transcriptional activation through the E2F (including EZH2) pathway to drive melanoma metastasis [28]. ZNF471 suppresses gastric cancer by transcriptionally repressing the PLS3 and TFAP2A downstream oncogenes [29]. Although several studies have revealed the expression level of TFAP2A and its impact in lung cancer based on single-cell RNA-seq analysis and bioinformatics analysis, TFAP2A may be involved in tumour progression [30, 31]. However, the mechanism by which TFAP2A promotes NSCLC progression has not been reported. The present study demonstrated that TFAP2A overexpression promoted tumour proliferation, invasion, and migration. In addition, TFAP2A may target ESR2 to activate MAPK signalling pathways and influence the effect of osimertinib.

Oestrogen plays a crucial role in the regulation of several physiological processes, including cell proliferation, development, and differentiation. Currently, the conventional oestrogen receptor (ER) is classified into two distinct subtypes, namely, ERα and ESR2. Research has indicated that oestrogen receptors play a significant role in the development of oestrogen-responsive cancers, including breast cancer, ovarian cancer and endometrial cancer. Oestrogen receptors are also expressed in cancers of various nontarget organs, such as liver cancer, gastric cancer, lung cancer and colorectal cancer. This finding suggests a potential association between oestrogen receptors and the progression of tumours in oestrogen nontarget organs. According to previous studies, ESR2 is the predominant oestrogen receptor in lung cancer [32]. However, the role of ESR2 in lung cancer remains uncertain, and the oncogenic role of ESR2 is a current topic in research [32–34]. Some researchers have suggested that ESR2 decreases the survival of NSCLC cells by regulating oncogenic RAS signalling [35]. These results may be caused by many factors, including the complex regulatory role of ESR2 in humans.

## Conclusion

In summary, the present study revealed that TFAP2A promotes NSCLC progression by mediating ESR2 and subsequently activating MAPK signalling pathways, and combined treatment with the ESR2 inhibitor TPHPP and targeted therapy synergistically inhibits tumour growth and improved patient prognosis.

## Materials and methods

### Cell culture, lentiviral transduction, cell transfection, and luciferase reporter assays

Human NSCLC cell lines (A549, H1299, H1703, H460 and H1975) were purchased from the cell library of the Chinese Academy of Sciences. All cells were cultured in DMEM/RPMI-1640 (Gibco, USA) supplemented with 10% FBS (Gibco, USA) and 2% penicillin‒streptomycin solution (YEASEN, China). A TFAP2A lentiviral vector was constructed (OBIO, China), and stably transfected cells were characterised by qRT‒PCR and Western blot analyses. siRNA oligonucleotides for ESR2 were designed, and transient transfection of cells was performed using Lipofectamine 2000 reagent for 48 h according to the manufacturer’s instructions (Invitrogen, USA). pGL3-TFAP2A (WT and mutant type)-LUC was constructed and cotransfected into HEK293T cells along with an ESR2 overexpression plasmid for 48 h. The cells were lysed, and the luciferase reporter activity was quantified utilising the Luciferase Reporter System (Promega, USA). The firefly luciferase levels were normalised to the Renilla luciferase levels. The targets of shTFAP2A and siESR2 are presented in Supplementary Table 1.

### qRT‒PCR and Western blot analyses

qRT‒PCR and Western blot analyses were performed according to previously described protocols [36], and the details are provided in the Supplementary Methods and Materials. The primers used are listed in Supplementary Table 2. The antibodies used are listed in Supplementary Table 3, and all reagents used are listed in Supplementary Table 4.

### Tissue samples and ethics

A total of 102 paired tumour and para-tumour cancer tissues were obtained from the Second Affiliated Hospital of Nanchang University and Fudan University Zhongshan Hospital, and the tissues were constructed into a tissue microarray (TMA). Patient information was collected from 2010 to 2012. All of the patients in the present study signed consent forms. The Ethics Committee of The Second Affiliated Hospital of Nanchang University and Fudan University Zhongshan Hospital granted approval for the procedures pertaining to human subjects.

### TMA and immunohistochemistry (IHC)

The TMA contained 102 paired tumour and para-tumour cancer tissues, and the TMA was used to perform IHC. In brief, the TMA was placed on a tissue sample slide dryer at 70 °C for 90 min (KEDEE, China), sequentially deparaffinized with xylene, and rehydrated with graded alcohol (anhydrous ethanol to 75% ethanol). Following the citrate/EDTA antigen retrieval method (Absin, China), the tissues were subjected to staining using an IHC detection kit (ZSGB-Bio, China). Two skilled pathologists evaluated the same IHC images. Positive staining was indicated by brownish yellow granules on the cell nucleus and/or cytoplasm, and semiquantitative methods were used to assess the percentage of positive cells and staining intensity under the microscope. To count the number of positively stained cells, five high-power fields (200×) on each section were observed, and the percentage of positively stained cells was determined. The scores for the number of positively stained cells were based on the percentage of positive cells as follows: <5%, 0 points; 5%≥ to < to 25%, 1 point; 25%≥ to <50%, 2 points; 50%≥ to <75%, 3 points; and 75%%≥ to ≤100%, 4 points. Positive staining intensity was scored as follows: 0 points for colourless, 1 point for light yellow, 2 points for brownish yellow, and 3 points for brownish brown. The multiplication of the two scores results in a positive rating as follows: 0 is negative (−), 1–4 is weakly positive (+), 5–8 is positive (++), and 9–12 is strongly positive (+++). The primary antibodies used are listed in Supplementary Table 3.

### CCK8, colony formation, wound-healing, and invasion assays

These assays were performed according to previously described methods [37, 38], and the details are provided in the Supplementary Methods and Materials.

### Available data from public databases

The expression level of TFAP2A in multiple cancers was obtained from TIMER 2.0. The bubble plot shown in Fig. 1B, the survival probability data shown in Fig. 1H, and the TFAP2A levels in LUAD and LUSC patients were downloaded from Sangerbox 3.0. The bubble plot was generated with R, and the detailed source code is provided in Supplementary file 1. Overall survival (OS) data were downloaded from the Kaplan–Meier plotter database. The data shown in Fig. 1G were supplied by TCGA. The relationships between TFAP2A and proliferative markers were analysed via the GEPIA 2 database. The GO, KEGG and GSEA data were downloaded from TCGA (TCGA-LUAD and TCGA-LUSC, https://portal.gdc.cancer.gov/) and analysed using R, and the detailed source code is provided in Supplementary file 2.

### Chromatin immunoprecipitation (ChIP)

A ChIP assay was performed to assess the binding region of TFAP2A in ESR2 using a ChIP kit (CST, USA) according to the manufacturer’s protocol. In brief, cells were crosslinked in 1% formaldehyde, and the DNA was sonicated to 200–1000 bp fragments. The ESR2/IgG antibody (Proteintech, China) was incubated with the mixture overnight, and the precipitated DNA was purified with ChIP-Grade Protein G magnetic beads (CST, USA). The target genes were quantified by qRT‒PCR.

### In vivo assays

The Animal Ethics Committee of Fudan University Zhongshan Hospital approved all the animal experiments. BALB/c nude mice aged 4–6 weeks were purchased from Jiesijie (Shanghai, China). All mice were maintained in an SPF-rated animal facility and underwent daily health checks. For the xenograft mouse model, 5 × 10^6^ A549-shCtrl, shTFAP2A-1, shTFAP2A-2, or A549-vector/TFAP2A cells were injected subcutaneously into the mice. The tumour size was measured every 6 days, and the mice were euthanized after 3 to 4 weeks. For drug administration, A549-TFAP2A cells were subcutaneously injected. After 6 days of tumour growth and development, the mice were randomly divided into four groups and treated every other day with an intraperitoneal injection of DMSO, osimertinib (10 mg/kg), PHTPP (4 mg/kg), or osimertinib (10 mg/kg) + PHTPP (4 mg/kg). The tumour was removed after 24 days.

### Statistical analysis

All the data were analysed by SPSS 27 and GraphPad Prism 9, and each experiment was repeated a minimum of three times. The data are presented as the mean ± standard deviation. Comparisons between two groups were conducted using either the t test or chi-square test, as appropriate. Multiple tests were repeated with one-way ANOVA or post hoc multiple tests. Survival analysis for the TFAP2A^High^ and TFAP2A^Low^ groups was conducted using Kaplan–Meier estimators, and group comparisons were made utilising log-rank tests. Univariate and multivariate Cox regression analyses were used to identify risk factors for NSCLC. Spearman correlation analysis was conducted to determine the correlation coefficients between TFAP2A and ESR2. *P* < 0.05 was considered to indicate statistical significance.

## Supplementary information

Supplementary Figure 1

Supplementary Materials

## Data Availability

All data generated or analysed during this study are included either in this article or in the supplementary information files.
