# Supplementary figures and images for: TFAP2A drives non-small cell lung cancer (NSCLC) progression and resistance to targeted therapy by facilitating the ESR2-mediated MAPK pathway
Source: Cell Death Discov. 2024 Dec 18;10:491. doi: 10.1038/s41420-024-02251-5 (PMC11655566; doi:10.1038/s41420-024-02251-5)

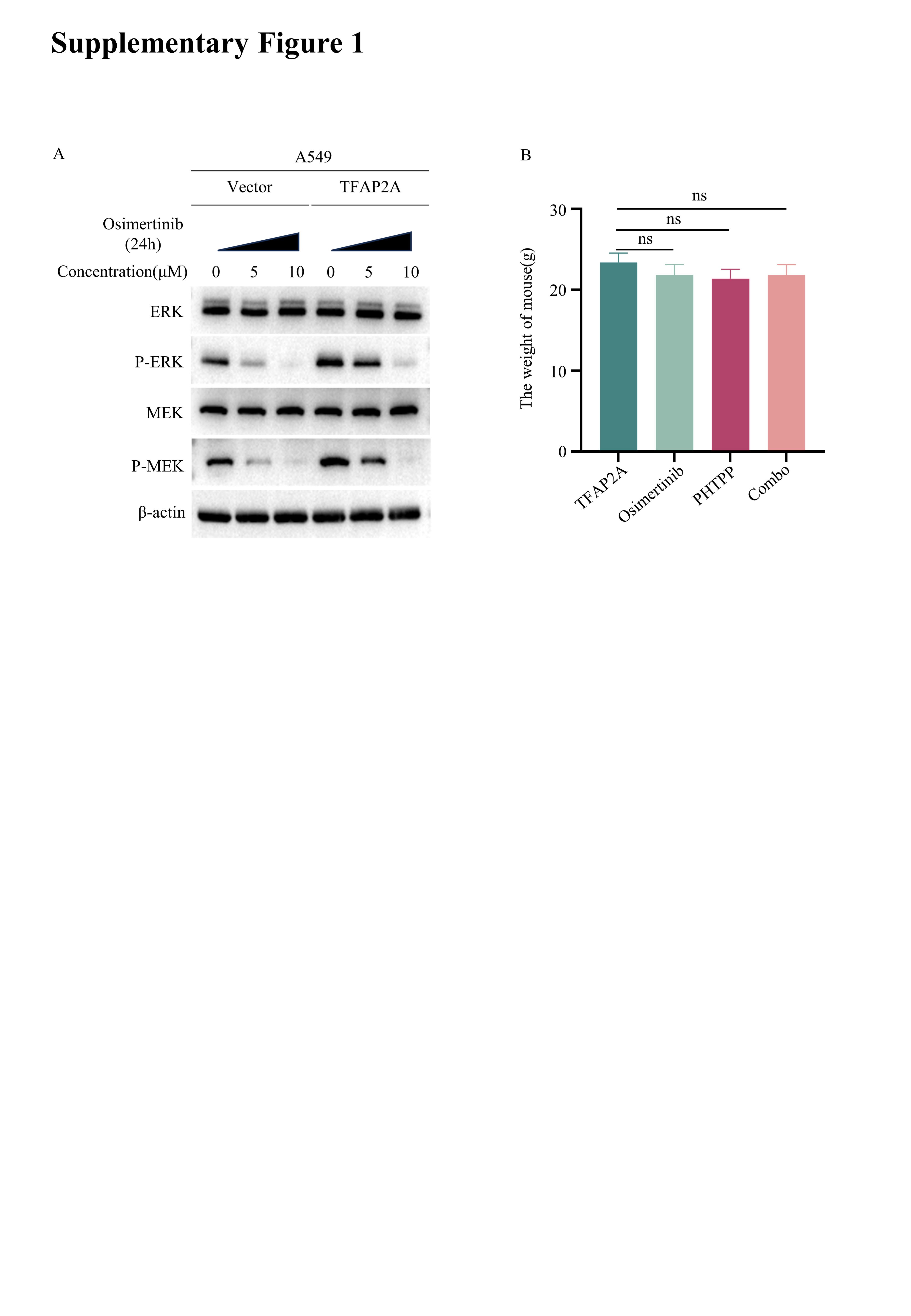

Supplement: Supplementary file 1 — Supplementary Figure 1 [file 41420_2024_2251_MOESM1_ESM.jpg]
