# Supplementary material for: TFAP2A drives non-small cell lung cancer (NSCLC) progression and resistance to targeted therapy by facilitating the ESR2-mediated MAPK pathway
Source: Cell Death Discov. 2024 Dec 18;10:491. doi: 10.1038/s41420-024-02251-5 (PMC11655566; doi:10.1038/s41420-024-02251-5)
Supplement: Supplementary file 2 — Supplementary Materials [file 41420_2024_2251_MOESM2_ESM.pdf]

Supplementary Methods and Materials

qRT–PCR and Western Blotting

RNA was extracted from cell lines and tissues; all steps were performed in accordance with the manufacturer's instructions. In brief, for reverse transcription, we mixed the total RNA, RT SuperMix, gDNA Remover Mix, and RNase-free water in a 20-μl final volume in an RNase-free centrifuge tube. Then, the first cDNA was obtained by a reverse transcription procedure: 37 °C for 5 minutes and 85 °C for 30 seconds. Thus, a qPCR assay was performed using Hieff® qPCR SYBR Green Master Mix (YEASEN, China) with a two-step procedure: 95 ℃, 5 minutes for 1 cycle, 95 ℃, 10 s and 60 ℃, 30 s for 40 cycles.

For Western blotting, the protein of cells and tissues was isolated and boiled with protein loading buffer (YEASEN, China) for 10 minutes at 100 ℃. Thus, the process of protein separation was conducted using SDS–PAGE, followed by the subsequent transfer of the isolated proteins onto PVDF membranes (Millipore, USA). The PVDF membranes were soaked in protein-free rapid blocking buffer for 15 minutes and incubated with primary antibody overnight at 4 ℃ and secondary antibody for 1 h at room temperature.

CCK8, colony formation, wound healing, and invasion assays

CCK8 and colony formation assays were used to assess the proliferation of cells. CCK8 was performed via a CCK-8 kit (YEASEN, China). We prepared 1000 cells with 100 μl of DMEM per well of a 96-well plate, 10 μl of CCK-8 reagent was added to each well for 2 h, and the absorbance was measured at a wavelength of 450 nm. All these steps were repeated after 24 h, 48 h, and 72 h. The dose of Osimertinib and PHTPP were 7.4 μM and 100nM.

For colony formation assays, approximately 2000 cells were placed in each well of 6-well plates (Corning, USA) with 3 ml medium for 14 days, and then cell colony numbers were fixed with 4% paraformaldehyde and stained with 0.4% crystal violet. The cells were cultured in 6-well plates for 24 h, and we used a 10 μl sterile pipette tip to draw a vertical line in the middle area of each well. Photos were taken at 0 h, 24 h, and 48 h. The results were analyzed by ImageJ.

For the invasion assay, we used 24-well plates equipped with chamber inserts featuring a pore size of 8 μm. A total of 2×10^4 cells in 200 μL medium without serum were added after the Matrigel was put into the upper chamber, and 500 μL medium with 10% FBS was put into the lower chamber. The cells were cultured for 36 h at 37 °C, fixed with 4% paraformaldehyde and stained with 0.4% crystal violet.

Supplementary Figure legends

Supplementary Figure 1

A Osimertinib reduced MEK and ERK phosphorylation at 0, 5, and 10 μM. **B** Weights of the mice in the four groups (TFAP2A, Osimertinib, PHTPP, and Combo).

Supplementary table 1. Target sequences of shRNA and siRNA used in this study

shRNA and siRNA	target sequence (5'→3')
shTFAP2A-1	ACAGAAGGAGGAAACACCAAT
shTFAP2A-3	TTATATCCACAGAAGGAGGAA
siERβ-1	TGCTTTGGTTTGGGTGATT
siERβ-2	GCCCUGCUGUGAUGAAUUA

Supplementary Table 2. The qRT-PCR primers used in this study

Gene	Forward primer (5'-3')	Reverse primer (5'-3')
TFAP2A	TTACTCCCACGTCAACGACC	GGTCTTCTACATGCGGGACC
ERβ	CAAGCTCATCTTTGCTCCAGA	GCCTTGACACAGAGATATTCTTTG
RARA	GGCGGAAGAAGCCCTTGCAG	CAGCCCTCACAGGCGCTGAC
ITPR2	AAAGCCTCAGTGGAATCCTGT	ATGGCAATTCCACGATTTTT
ADCY6	CAGCAGGGTAGTGTGTGCAG	TCTGCATTTGATTTTGGCCT
GAPDH	GGAGCGAGATCCCTCCAAAAT	GGCTGTTGTCATACTTCTCATGG

Supplementary table 3. Antibody for western blotting, RIP and IHC

**Antibody**	**Company**	**Cat No.**
TFAP2A	Abcam	ab108311
ESR2	Proteintech	14007-1-AP
MEK1/2	CST	9122S
p-MEK1/2	CST	9154S
ERK1/2	CST	4695S
p-ERK1/2	CST	4370S
ERK1/2	proteintech	51068-1-AP
p-ERK1/2	proteintech	28733-1-AP
GAPDH	Proteintech	60004-1-Ig
IgG	Abcam	ab172730
Peroxidase AffiniPure Goat Anti-Rabbit lgG(H+L)	YEASEN	33101ES60
Peroxidase AffiniPure Goat Anti-Mouse IgG(H+L)	YEASEN	33201ES60

Supplementary table 4. The relevant reagents

Reagents	Company	Cat No.
Tris-Glycine SDS-PAGE Running Buffer (Powder)	Servicebio	G2018-15
Tris-Glycine Transfer Buffer (Powder)	Servicebio	G2017-15
Primary Antibody Dilution Buffer for Western Blot	Epizyme	PS114
Secondary Antibody Dilution Buffer for Western Blot	Epizyme	PS115
qPCR SYBR Green Master Mix(No Rox)	YEASEN	11201ES08
Hifair® AdvanceFast One-step RT-gDNA Digestion SuperMix for qPCR	YEASEN	11151ES60
Cell lysis buffer for Western and IP	Beyotime	P0013
Protease inhibitor cocktail for general use, MS-SAFE, 50X	Beyotime	P1008

Supplementary file 1

```
getwd()
setwd('D:\\NCU\\R\\TFAP2A')
df<-read.csv("TFAP2A qipao.csv",header=T)
head(df)
library(ggplot2)
ggplot(df,aes(number,expression,size=pvalue))+
  geom_point(aes(size=pvalue,color=group))
```

Supplementary file 2

```
library("org.Hs.eg.db")
library("clusterProfiler")
library("enrichplot")
library("ggplot2")
library("ggnewscale")
library("enrichplot")
library("DOSE")
library(stringr)

pvalueFilter=0.05
qvalueFilter=1
showNum=8

rt=read.table("target.txt",sep="\t",check.names=F,header=F)
genes=as.vector(rt[,1])
entrezIDs <- mget(genes, org.Hs.egSYMBOL2EG, ifnotfound=NA)
entrezIDs <- as.character(entrezIDs)
rt=cbind(rt,entrezID=entrezIDs)
colnames(rt)=c("symbol","entrezID")
rt=rt[is.na(rt[,"entrezID"])==F,]
gene=rt$entrezID
gene=unique(gene)

colorSel="qvalue"
if(qvalueFilter>0.05){
   colorSel="pvalue"
}


kk=enrichGO(gene = gene,OrgDb = org.Hs.eg.db, pvalueCutoff =1, qvalueCutoff = 1, ont="all", readable =T)
GO=as.data.frame(kk)
GO=GO[(GO$pvalue<pvalueFilter & GO$qvalue<qvalueFilter),]

write.table(GO,file="GO.xls",sep="\t",quote=F,row.names = F)

library("clusterProfiler")
library("org.Hs.eg.db")
library("enrichplot")
library("ggplot2")
library("pathview")
library("ggnewscale")
```

```
library("DOSE")
library(stringr)

pvalueFilter=0.05
qvalueFilter=1
showNum=20
keggId="hsa04659"

rt=read.table("target.txt",sep="\t",check.names=F,header=F)
genes=as.vector(rt[,1])
entrezIDs <- mget(genes, org.Hs.egSYMBOL2EG, ifnotfound=NA)
entrezIDs <- as.character(entrezIDs)
rt=cbind(rt,entrezID=entrezIDs)
colnames(rt)=c("symbol","entrezID")
rt=rt[is.na(rt[,"entrezID"])==F,]
gene=rt$entrezID
gene=unique(gene)
colorSel="qvalue"
if(qvalueFilter>0.05){
	colorSel="pvalue"
}
kk <- enrichKEGG(gene = gene, organism = "hsa", pvalueCutoff =1, qvalueCutoff =1)
KEGG=as.data.frame(kk)
KEGG$geneID=as.character(sapply(KEGG$geneID,function(x)paste(rt$symbol[match(strsplit(x,"
/")[[1]],as.character(rt$entrezID))],collapse="/")))
KEGG=KEGG[(KEGG$pvalue<pvalueFilter & KEGG$qvalue<qvalueFilter),]

write.table(KEGG,file="KEGG.xls",sep="\t",quote=F,row.names = F)

setwd("D:\\NCU\\R\\tumour\\LUAD\\GSEA")
library(org.Hs.eg.db)
library(clusterProfiler)
library(pathview)
library(enrichplot)
library(dplyr)
library(reshape2)
library(ggplot2)
library(ggridges)
library(biomaRt)
library(rlang)
data <- read.csv("GSEA DIFFER.csv")
colnames(data)[8]="SYMBOL"
head(data)
gene = data$SYMBOL
```

```
gene=bitr(gene,fromType="SYMBOL",toType="ENTREZID",OrgDb="org.Hs.eg.db")
gene = dplyr::distinct(gene,SYMBOL,.keep_all=T)
data_all <- data %>%
  inner_join(gene,by="SYMBOL")
data_all_sort <- data_all %>%
  arrange(desc(log2FoldChange))
geneList = data_all_sort$log2FoldChange
names(geneList) <- data_all_sort$ENTREZID
KEGG_database="hsa"
gsea <- gseKEGG(geneList, organism = KEGG_database, pvalueCutoff = 0.05)
gsea <- setReadable(gsea, OrgDb=org.Hs.eg.db,keyType = 'ENTREZID')
dotplot(gsea) #有问题报错：Error in .standalone_types_check_dot_call(ffi_standalone_check_number_1.0.7, :
  object 'ffi_standalone_check_number_1.0.7' not found
ridgeplot(gsea,label_format = 100)
gseaplot2(gsea,1,pvalue_table = T)
write.csv(gsea,file="GSEA RESULTS.csv", quote = F) #输出文件
```

Fig 2B

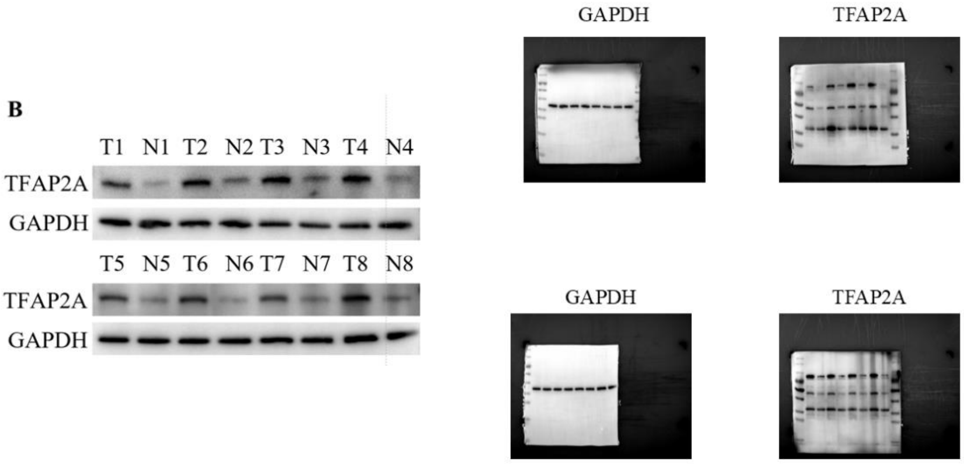

Fig 3B

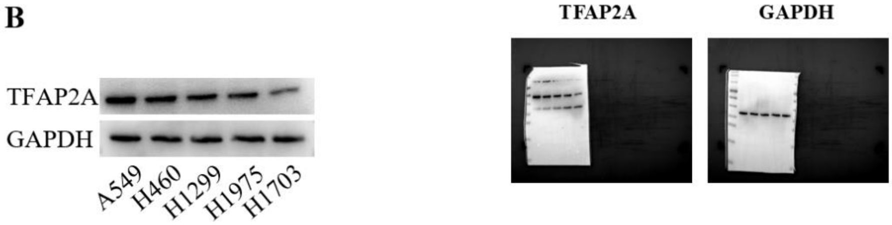

Fig 3C

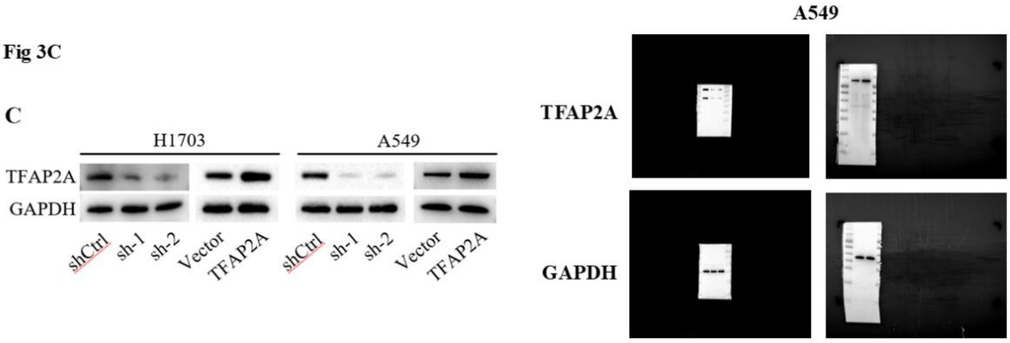

Fig 3C

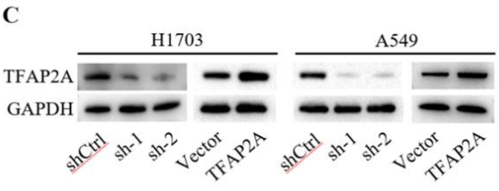

H1703

FAP2A

;APDH

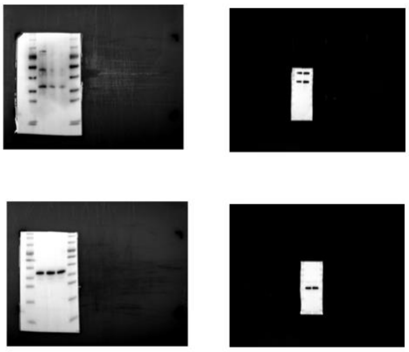

Fig 5F

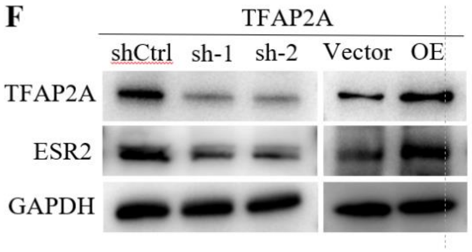

TFAP2A

ESR2

GAPDH

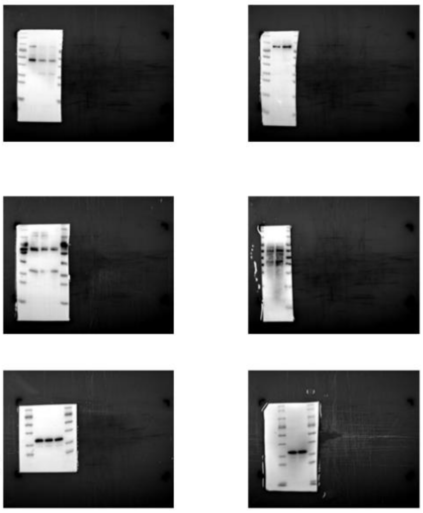

Fig 6A

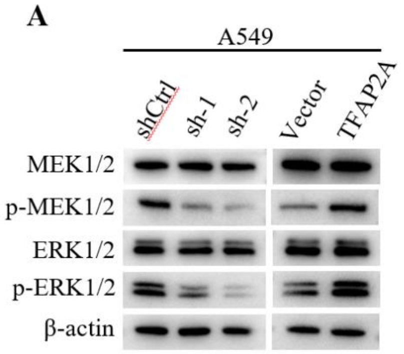
A
A549
shCtrl
sh-1
sh-2
Vector
TFAP2A
MEK1/2
p-MEK1/2
ERK1/2
p-ERK1/2
β-actin

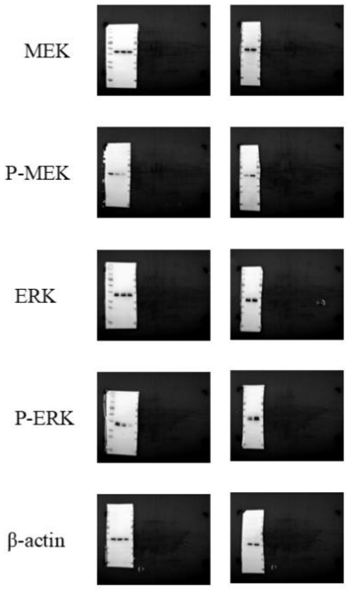
MEK
P-MEK
ERK
P-ERK
β-actin

Fig 6D

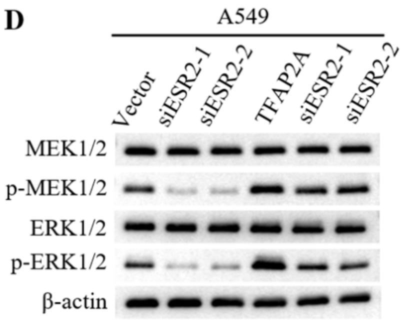
D
A549
Vector
siESR2-1
siESR2-2
TFAP2A
siESR2-1
siESR2-2
MEK1/2
p-MEK1/2
ERK1/2
p-ERK1/2
β-actin

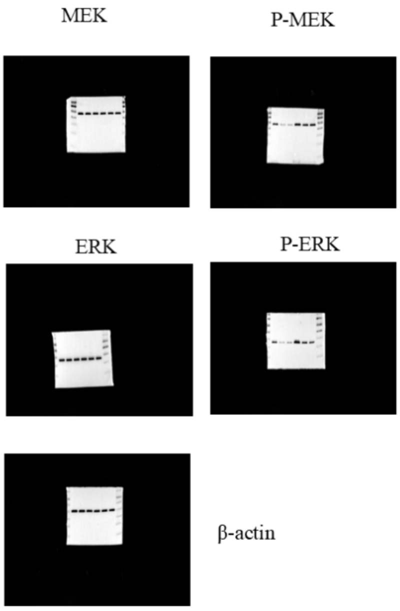
MEK
P-MEK
ERK
P-ERK
β-actin

Supplementary Fig 1

A

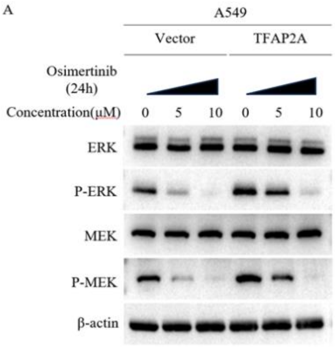

MEK

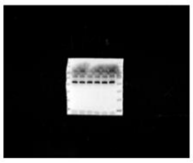

P-MEK

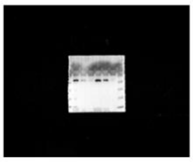

ERK

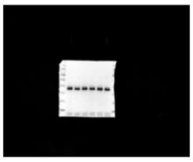

P-ERK

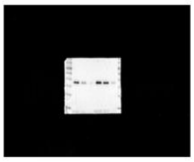

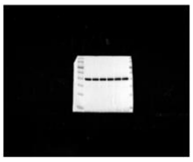

β-actin
